# The Effects of Population Aging on Sports Industry Development: The Mediating Effect of Technological Innovation

**DOI:** 10.3390/ijerph20032085

**Published:** 2023-01-23

**Authors:** Xiaolong Wei, Tao Wang, Yang Chen, Oleksii Lyulyov, Tetyana Pimonenko

**Affiliations:** 1 School of Sports Science, Fujian Normal University, Fuzhou 350117, China; 2 School of Economics, Fujian Normal University, Fuzhou 350117, China; 3 Department of Management, Faculty of Applied Sciences, WSB University, 41-300 Dabrowa Gornicza, Poland; 4 Department of Marketing, Sumy State University, 40007 Sumy, Ukraine

**Keywords:** income inequality, education, aging population, sports industry, technological innovation

## Abstract

It is of great practical significance to rationally formulate a development strategy for the sports industry to deeply understand and comprehensively grasp the impact of population aging on the development of the sports industry. To study the impact of population aging on the development of the sports industry, panel data at the provincial level in China from 2014 to 2020 are selected, and a mediation effect model is established to test how the aging of the population affects the development of the sports industry through technological innovation. The results show that technological innovation can explain 59.87% of the impact of population aging on the development of the sports industry without considering the control variables, and the impact of population aging on labor productivity through technological innovation is positive. Under the condition of considering the control variables, technological innovation can explain 56.74% of the impact of population aging on the development of the sports industry, and the impact of population aging on the development of the sports industry through technological innovation is positive. The proportion of the population aged 65 and above in the total population was used as a proxy variable for population aging to test the robustness test, and the impact of technological innovation on the development of the sports industry was positive without considering the control variables. In the robustness test considering the control variables, the impact of technological innovation on the development of the sports industry is positive.

## 1. Introduction

Since the 18th CPC National Congress of the Communist Party of China, China’s economic development has shifted from low-cost labor and other factors to innovation-driven investment. The report to the 19th National Congress of the CPC pointed out that innovation is the primary driving force for economic development and the strategic support for building a modernized economic system. The party’s 20th annual report noted that we will continue to accelerate the implementation of the innovation-driven development strategy to promote China’s economic development. As an important part of the national economy, the sports industry must rely on the coordinated upgrading of the basic factors of production to realize the development of population and technology. It is worth noting that any hindered development is not conducive to the development of the sports industry. In recent years, China’s population development has entered a new normal, that is, a reduction in the population growth rate, a reduction in the working-age population, accelerated population aging, and an improvement in population quality. Among them, population aging, as a structural result of population transformation, has the irreversible and continuous deepening characteristics of [1,2], which makes the development of China’s sports industry face the unfavorable situation of “getting old before getting rich” [3]. The aging of the population will cause a series of economic and social problems, such as insufficient labor supply and declining savings rates, which will then weaken [4] sports consumption demand and weaken the growth momentum of the sports industry. In view of the background of the continuous deepening of population aging, on the one hand, academic circles believe that the innovation-driven development of the sports industry should be promoted by improving the technological innovation ability of enterprises to improve the total factor productivity of the sports industry. On the other hand, the Chinese government has also formulated the National Medium- and Long-term Plan for Actively Responding to Aging and proposed actively responding to population aging from five aspects, including scientific and technological innovation ability, clearly implementing the innovation-driven development strategy, and taking technological innovation as the first driving force and strategic support to actively respond to the population aging population.

Technological innovation can not only promote the development of the sports industry as a driving force but also provide strategic support for coping with the aging of the population. What impact will the aging of the population have on technological innovation? How can population aging develop the sports industry by restricting technological innovation? There are two completely opposing views on the sports field. First, from the perspective of the whole life cycle, the aging of the population will hinder technological innovation and then have a negative impact on the development of the sports industry. With increasing age, the elderly’s physical skills, cognitive ability, and subjective innovation motivation weaken [5], directly or indirectly hindering technological innovation and leading to the development of the sports industry [6]. Second, from the perspective of human capital [7,8,9] accumulation, population aging will promote technological innovation and then promote the development of the sports industry. The increasing aging of the population means that the elderly population is in good health, with a longer and higher level of education [10] and much work experience, which helps to improve technological innovation and improve the productivity of the sports industry [11]. The above study is mainly based on the microlevel analysis of the impact of population aging on technological innovation, and its conclusions are not fully applicable to infer the impact of macrolevel population aging on technological innovation. In addition, the sports community has also made some contributions to the impact of population aging on the development of the sports industry, which is mainly divided into three aspects. First, the aging population weakens residents’ sports consumption demand, which adversely affects [12] the “virtuous cycle” of the sports industry. Second, the increase in the elderly population is conducive to the development of the sports industry [13]. Finally, the integration of the sports industry and pension industry can be traced back to cope with the increasing population aging [14]. In summary, the existing research results in the sports field have paid attention to the impact of population aging on the sports industry, but the factors of population aging and how to affect the development of the sports industry have not yet appeared. Specifically, a simple experience description of the aging structure of the population and the sports industry has not effectively revealed the impact mechanism of aging on technological innovation, and what ways and mechanisms of aging impact technological innovation is still an incompletely understood empirical problem in the field of athletics [15].

Therefore, in the critical period of the transformation of the development power mechanism of China’s sports industry, it is necessary to take precautions for the aging of the population and formulate a long-term development strategy for the sports industry under the condition of population aging as early as possible. Therefore, it is of great significance to explore whether the aging population will lead to technological innovation, which is not conducive to the development of the sports industry. Based on the analysis of previous relevant literature, this paper fills the theoretical gaps in the assessment of (1) the impact of population aging on technological innovation and sports industry development and (2) the mediating effect of technological innovation in the chain “population aging–sports industry development”.

## 2. Literature Review

Because the decisive factors of the healthy and sustainable development of the sports industry are labor, capital, and technology, population aging will have an important impact on these three factors. Among them, technological innovation is the most important influencing factor among the three. The transformation of the technologically innovative production mode can not only alleviate the aging of the population but also be a factor in the transformation of the development mode of the sports industry.

### 2.1. The Relationship between Population Aging and Technological Innovation

According to the above elaboration, technological innovation is the most important intermediary variable in the influence of aging on the development of the sports industry. Many scholars guided [16] by internal student growth theory, believing that population aging affects technological innovation by affecting the accumulation of human capital and R&D investment [17,18,19,20], thus, affecting the healthy and sustainable development of the sports industry, but most research conclusions are not consistent. From the individual level, some scholars believe that the decline of physical function and cognitive ability causes the decline of individual innovation ability and then affects technological innovation [21]; others believe that the rich work experience of the elderly population has higher productivity compared with their younger counterparts, which is conducive to the realization of technological innovation [22]. At the enterprise level, some scholars believe that the aging population leads to rising labor costs and social security, which causes a “crowding out effect” on R&D investment, which is not conducive to technological innovation [23,24,25]; others believe that aging enterprises will fail to carry out technological innovation and then have a positive impact on enterprise technological innovation [26]. At the government level, some scholars believe that the aging population cannot provide a good policy environment for technological innovation, which hinders technological innovation [27] to some extent; others believe that the aging population will reduce the input of labor factors, but the increase in high-quality human capital will promote technological innovation [28]. The impact of population aging on technological innovation is relatively complex, both positive and negative, and the final impact is the result of the offset of the two effects. In addition, some scholars believe that the impact of population aging on technological innovation is not a nonnegative or negative binary opposition result but a “nonlinear” impact [29]. That is, in the early stage of aging, as society as a whole attaches great importance to human capital investment and technological innovation, the positive effect of technological innovation is far greater than the negative effect. The aging of the population has a positive impact on technological innovation. In the middle stage of aging, given the increasingly negative impact of the pension burden on technological innovation, combined with the slowdown of human capital accumulation and technological innovation caused by aging, the promoting effect of population aging on promoting technological innovation decreases [30]. In the later stages of aging, as the negative impact of the pension burden on technological innovation reaches its limit, the accumulation of human capital and technological innovation caused by aging has negative impacts on technological innovation [31].

### 2.2. The Relationship between Technological Innovation and the Development of the Sports Industry

Technological innovation is the core element to realize the development of the sports industry, and its impact on the development of the sports industry is not a simple linear relationship, which can not only promote the development of the sports industry but also inhibit its development. First, there are many ways for technological innovation to drive the development of the sports industry, which can usually play a positive role in various forms, such as product innovation and production process innovation, which is reflected in both industrial upgrading and achievement sharing. First, technological innovation catalyzes the application of new processes, new products, and new materials in the sports industry and then causes the change in the production demand structure and consumer demand structure to be [32] to drive the optimization and upgrading of the sports industry structure. Second, technological innovation improves sports industry productivity and input–output levels, reduces the cost and price of sports products, catalyzes the emergence of new sports products, increases the number of sports product consumption and consumption types, and optimizes consumption choices to effectively promote the development of sports industry achievement sharing and promote the coordinated development of the sports industry [33]. In addition, technological innovation can enhance the technological progress of sports enterprises and promote the transformation of the sports industry’s scientific research theory into practical achievements to improve the total factor production efficiency of the sports industry and expand its production boundary. Second, technological innovation hinders the development of the sports industry, mainly reflected in the two aspects of economic benefits and achievement sharing. First, the technological innovation applied in sports industry production brings new sports products, but in the early stages, misleading the capital market for new sports products produces high expectations and the risk of false prosperity. At the same time, a large influx of capital enhances the risk of excess capacity [34], and both superpositions may inhibit sports industry development. Second, technological innovation to speed up the process of eliminating the backward sports industry will increase the demand for specific workers and gradually eliminate incompetent workers, which is the result of income distribution bias to specific skills of workers, resulting in the income gap and widening the gap between rich and poor individuals and sports industry development, especially the benign cycle of negative effects.

### 2.3. The Relationship between Population Aging and The Development of the Sports Industry

Research on the impact of population aging on the development of the sports industry is still in its initial stage. The essence of the development of the sports industry lies in improving the growth quality of the sports industry at the core. In view of this, the aging of the population mainly affects the high-quality development of the sports industry from the perspective of supply and demand. First, from the supply side. The aging of the population leads to a decline in the size and proportion of the working-age population and a decrease in the labor supply, which not only suppresses the sports consumption level [35] of the whole society but also limits the improvement of labor productivity in the sports industry. Of course, the decrease in labor supply and rising labor costs caused by the aging of the population may also force the use of capital and technology to replace labor, improve labor productivity, and promote the development of the sports industry [14]. In addition, the aging population is not conducive to the accumulation of individual human capital and public human capital, thus, hindering the development of the sports industry. However, it is undeniable that the aging of the population also increases the investment [36] of individual and public human capital through the extension of life expectancy, thus, promoting the development of the sports industry. Second, on the demand side. The aging of the population brings about the rise or decline of the savings rate, which depends on the health degree of the population and the population’s life expectancy of [37] and then has a positive or negative impact on the consumption structure of [38], resulting in the development or regression of the sports industry. The intuitive result of population aging is the rapid growth of the elderly population, resulting in a huge aging sports industry market [39], but the aging sports industry in China is in its infancy. The contribution to the development of the sports industry in China will gradually rise [40], which will also to a certain extent promote the development of a high-quality economy in our country. In summary, the internal mechanism of population aging affecting the development of the sports industry is mainly realized through the impact of population aging on the development factors of the sports industry (labor supply, human capital, savings rate, and consumption). In this way, different combinations of sports industry development elements will produce different sports industry development modes and then directly act on the quality and effect of the sports industry, so it can be inferred that population aging has a significant impact on the development of the sports industry.

In summary, different scholars have found that the role mechanism of population aging and technological innovation in the development of the sports industry is nonlinear, with both positive and negative effects. At the same time, there is still some space for expansion in the above research. First, the existing results mainly focus on the study of the relationship between population aging and sports industry development, technological innovation and sports industry development, and population aging and technological innovation, which lacks the internal logical connection between the three. Second, China’s sports industry has entered a new stage of development, and its development power, development mode, and development goals have undergone important changes, but there are few studies on the relationship between the new stage. Considering the above, the study examines the following hypothesis:

**Hypothesis** **1 (H1):** *Population aging statistically impacts technological innovation and sports industry development.*

**Hypothesis** **2 (H2):** *Technological innovation has a mediating effect on the chain of “population aging–sports industry development”.*

## 3. Materials and Methods

### 3.1. Data Source

In 2014, the State Council issued several Opinions on Accelerating the Development of the Sports Industry and Promoting Sports Consumption, which was considered by the sports circle as the time node for the rapid development of China’s sports industry. The object of investigation was limited by the available data on sports industry development. Thus, the study analyzes 21 provinces for 2014–2020. The data of this paper are mainly from the China Statistical Yearbook, the official websites of the provincial sports bureaus and bureaus of Statistics, the provincial sports development plans of the 12th and 13th Five-Year Plans, the official website of the General Administration of Sport of China and the Bureau of Statistics, and the statistical yearbooks of the provinces. To avoid endogeneity and multicollinearity, this paper adopts logarithmic treatment for the total output value of the sports industry, the dependency ratio of the elderly population, technological innovation, industrial structure, human capital, population age structure, openness to the outside world, urbanization level, and transportation infrastructure.

The dependent variable of this study is the total output value of the sports industry, the total achievement or the monetary performance of all the production activities of the sports industry units in a certain period of time, and an important indicator to measure the development of the sports industry. The core independent variables of this study were technological innovation and population aging. The literature uses patent applications, patent grants [41], and R&D personnel to measure the technology innovation level, considering the selective difference in patents granted to patents and regional approval link measurement error, and R&D personnel in the micro aspects constitute the lack of reliable data sources, so R&D funds are used to measure technology innovation. In this paper, the dependency ratio of the elderly population aged 65 and above is used to measure the aging of the population, and the robustness test uses the proportion of the population aged 65 and above to replace the proportion of the total population.

In addition to the core independent variables focused on in this study, industrial structure, the age structure of the population, human capital level, urbanization, openness, and transportation infrastructure were included as control variables in the empirical model. According to the first-Clark law, the current sports industry structure changes along the “primary industry-secondary industry-tertiary industry”. With the refinement of the social division of labor and the change in people’s demand, the capital-labor-commodity flow between sports industry departments is becoming increasingly complex, and the reconfiguration of factors of production is conducive to the development of the sports industry. In this study, the total value proportion of the tertiary industry and the secondary industry was used to measure the industrial structure. The larger the value is, the more advanced the industrial structure. Using the number of provincial college students per 100,000 to measure regional human capital, the aging of the population reduces the scale of the labor force, and the labor supply advantage gradually disappears. Whether from the macro or micro level, improving the level of human capital can neutralize the disadvantages of the disappearance of the population size advantage. Using the proportion of the urban population of the total population level of urbanization, the level of urbanization gathered the urban land area of sports industry activities; the higher the level of urbanization, the higher the level of sports industry activity, active sports industry activity month, knowledge spillover, labor, specialized input, capacity output, and other agglomeration effect is higher. The dependence of regional economic development on commodity imports and exports is used; that is, the total amount of imports and exports are used to measure openness. Import and export are motivations for economic growth. In an open economic environment, the degree of market openness depends on foreign trade, which plays an important role in promoting the transformation and upgrading of the sports industry. Transportation infrastructure is a necessary condition for the development of the regional sports industry. Convenient transportation infrastructure helps to promote the development of the regional sports industry [42], eliminate the barriers to factor flow, and promote labor, capital, information, and high value-added intermediate product element flow [43], so this paper adopts railway mileage length to measure the perfection of transportation infrastructure. The findings of descriptive statistics are shown in Table 1.

### 3.2. Model Setting

To consider whether technological innovation affects the total output value of the sports industry under the background of population aging, this study establishes an intermediary effect model to verify the sports industry development benefits of technological innovation. To reduce the estimation bias of the missing variables and ensure the robustness of the regression results, the panel fixed effect model is adopted as follows:

(1) $$TS_{it}=\varphi_{1}+\omega_{1}senior_{it}+\tau_{1}Y_{it}+\theta_{1}$$

(2) $$tr_{it}=\varphi_{2}+\omega_{2}senior_{it}+\tau_{2}Y_{it}+\theta_{2}$$

(3) $$TS_{it}=\varphi_{3}+\omega_{3}senior_{it}+\omega_{4}tr_{it}+\tau_{3}Y_{it}+\theta_{3}$$

where for $TS_{it},\;senior_{it},\;tr_{it},\;Y_{it},\;\theta_{1},\;\theta_{2},\;\theta_{3}$ *i* represents the sample box (21 provinces), and *t* represents the scope of practice (2014–2020), indicating that the total output value of the sports industry is an aging population, technological innovation, a control variable, and a random interference item. Formula (1) indicates the total effect of population aging on the total output value of the sports industry and measures the total effect of population aging; formula (2) the impact of population aging on intermediary variables; and (3) the direct effect of population aging on the total output value of the sports industry.$\omega_{1}$

Formula (2) substitutes formula (3) to derive the intermediary effect of population aging on the total output value of the sports industry:

(4) $$TS_{it}=(\varphi_{3}+\varphi_{2}\omega_{4})+(\omega_{3}+\omega_{2}\omega_{4})senior_{it}+\tau_{4}Y_{it}+\theta_{4}$$

$\omega_{2}\omega_{4}$—measure the intermediary effect of population aging on the total output value of the sports industry.

## 4. Results

To verify whether all the variables will have a collinearity problem, the VIF test is used (Table 2).

From Table 2, we can see that the VIF of all the variables does not exceed 10, and there is no collinearity problem for this. According to Table 3, the panel data model AR test was passed, and there was no sequence autocorrelation. The Sargan test was passed, and the instrumental variables were selected appropriately. Some studies have pointed out that the deepening of aging and the investment in scientific research experience hinders the technological innovation of countries, enterprises, and individuals at the macromedium-micro level and then affects the development of the sports industry [44]. However, as shown in Table 3, model (1) and model (2) are the benchmark models for the impact of population aging on the development of the sports industry and technological innovation, and model (3) is the technological innovation effect of population aging on the development of the sports industry.

The results of model (1) and model (2) show that population aging can significantly promote the development of the sports industry and the iteration of technological innovation, and the impact of population aging on the development of the sports industry and technological innovation iteration is positive, which also shows that a further intermediary effect test can be performed. The results of model (3) show that the aging of the population has a positive impact on the development of the sports industry, and technological innovation also has a positive impact on the development of the sports industry, indicating that the technological innovation effect of population aging on the development of the sports industry is significantly positive; that is, the aging of the population will promote the development of the sports industry by improving technological innovation.

When considering control variables, sequence autocorrelation and instrumental variable selection tests were passed. According to Table 3, under the constraints of other factors, model (4) and model (5) are the benchmark models for the impact of population aging on the development of the sports industry and technological innovation, and model (6) is the technological innovation effect of population aging on the development of the sports industry. The results of model (4) and model (5) show that population aging can significantly promote the development of the sports industry and the iteration of technological innovation, and the impact of population aging on the development of the sports industry and technological innovation iteration is positive, which also shows that a further intermediary effect test can be performed. The results of model (6) show that the aging of the population has a positive impact on the development of the sports industry, and technological innovation also has a positive impact on the development of the sports industry, indicating that the technological innovation effect of population aging on the development of the sports industry is significantly positive. That is, the aging of the population will promote the development of the sports industry by improving technological innovation.

In Table 3, model (4), model (5), and model (6) control variable results, industrial structure, human capital, opening to the outside world, transportation infrastructure, and the influence of technological innovation and sports industry development are significantly positive, and industrial structure, human capital, opening to the outside world, and transportation infrastructure through technological innovation significantly affect the development of the sports industry. However, the impact of urbanization on technological innovation and sports industry development is not significant.

To test the robustness of the regression results, the proportion of people aged 65 years and older in the total population was used to measure the aging degree of the dependency ratio of the elderly population. According to model (7), model (8), and model (9), the basic estimation results of the model were not changed after changing the measurement index of the main variable (Table 4).

The robustness results (Table 4) show that it can be reflected in two aspects. First, the overall effect of population aging on the development of the sports industry is still significantly positive, and it also has a significant role in promoting technological innovation. Second, population aging can significantly affect the development of the sports industry through technological innovation. This indicates the strong robustness of the estimated results, which further verifies the rationality of the above analysis.

## 5. Discussion

The findings showed that population aging impacted the development of the sports industry and technological innovation, and the technological innovation effect of the population aging effect on the development of the sports industry. The reason may be that China’s overall labor force is still large, which is supported by the relevant data of the seventh national population census; that is, the population aged 15–59 in China is 894.38 million, and the supply of the working-age population still has advantages. The advantage of the school-age labor force still does not squeeze out the scientific research funds of countries, regions, and enterprises. Still, it will help individuals invest a large number of funds in improving their human capital, promote the iteration of technological innovation, and promote the development of the sports industry. At the same time, the deepening population aging forms a forcing mechanism for technological innovation. Population aging is not only the aging of the entire population structure but also the aging of the productive population structure [45,46]. This is mainly because technological innovation can optimize the allocation structure of sports industry resources and improve the production efficiency of sports industry units under the condition of other sports production means [47,48,49].

In the process of deepening population aging, the positive externalities of the cumulative effect of human capital will be fed back to the level of sports industry enterprises, promoting sports industry enterprises to continuously increase investment in scientific research funds and then promote the development of the sports industry. In addition, from the perspective of economic growth theory, the increase in scientific research investment from countries, regions, enterprises, and individuals will inevitably promote the development of the sports industry [25]. In the context of population aging, technological innovation plays a role as an intermediary variable to promote the development of the sports industry, which was also verified in this study. Therefore, the increase in scientific research funds and the emergence of many technological innovations have played an important role in saving the labor cost of the sports industry. It allows for optimizing the structural allocation of sports industry resources, improving the unit output of the sports industry, and providing a feasible way to deal with aging actively [50,51].

It should be noted that after considering the control variables, the interpretation degree of the impact of population aging on the development of the sports industry through the technological innovation path is greatly improved. Some studies [52,53,54] show that in actual industrial development, population aging will harm technological innovation. Thus, a positive effect of some neutralization technological innovation on the development of the sports industry. However, in this paper, the aging population can promote the development of the sports industry, which may be due to the elderly population and the working-age population decreasing year by year, combined with the elderly population’s education level and health awareness. Furthermore, the aging population promotes the expansion of the sports consumption market and the adjustment of the sports consumption structure, thus, reversing the transmission sports industry’s continuous technological innovation. At the same time, the improvement of the average life expectancy will push some of the elderly population with rich working experience back into the sports industry market again, and these groups will promote the sustainable and healthy development of the sports industry through cumulative technological innovation. In addition, the control variables (industrial structure, age structure of the population, human capital level, urbanization, openness, and transportation infrastructure) show a positive impetus for technological innovation, especially the human capital elements. Combined with the changing trend of the increasing output value of China’s sports service industry from 2014 to 2020, the development of China’s sports industry began to shift from factor- and investment-driven to innovation-driven [55]. However, it cannot be ignored that because China’s sports industry started late and accounted for a relatively high proportion, the adjustment of the sports industry capital-labor allocation structure has a certain lag. In this case, it is necessary to start from the supply side to stimulate the power of technological innovation in sports industry enterprises and improve the unit labor output.

## 6. Conclusions

Against the background of increasing population aging, how to play the role of technological innovation in the sports industry is the key to the transformation of an innovative country. This paper empirically examines the functional mechanism of technological innovation in the sports industry from the perspective of population aging. First, technological innovation can explain 59.87% of the impact of population aging on the development of the sports industry, and the influence of population aging on labor productivity through technological innovation is positive. Technological innovation can explain 56.74% of the impact of control variables, and population aging is positive on the development of the sports industry through technological innovation. Second, the proportion of the population aged 65 years and above in the total population as an alternative variable for the aging of the population proves that the influence of technological innovation on the development of the sports industry is positive without considering the control variables, and the influence of technological innovation on the development of the sports industry is positive.

The transition from an industrial to a service-oriented structure has improved the development of the sports industry. From a macro perspective, this is mainly due to the increase in the contribution of China’s sports industry to economic growth year by year from 2014 to 2020. From the micro point of view, the proportion of the sports service industry gradually increased and gradually turned to the development direction of high value-added value to promote the sports industry structure in the advanced direction and effectively improve the output rate. Human capital is an important factor in improving technological innovation. Under the condition of fixed capital and labor resources, improving the human capital level improves the unit output efficiency of the sports industry capital and labor force. At the same time, the cumulative effect of human capital expands the structural allocation space of capital and labor force, and the allocation optimization and evolution of the two human capitals is also a process of improving the unit labor output of the sports industry. The role of opening to the outside world and transportation infrastructure promoted the sports industry at this stage. After the reform and opening up, China gradually expanded its opening to the outside world, foreign trade increased year by year, and the export trade of sports goods also increased year by year [56], among which the proportion of sports goods with advanced technology content also increased year by year, which shows that the volume of import and export trade promotes the development of China’s sports industry [57]. By 2020, China’s railway operating mileage was 146,300 km, ranking among the highest in the world. The improvement of the railway and other transportation foundations has greatly shortened the circulation of the means of production of the sports industry in the circulation link, improved its utilization rate, and played an important role in promoting the development of the sports industry. Fourth, the role of urbanization in developing the sports industry is not obvious at this stage. However, China has formed the Fujian province, Guangdong experience, and Jiangsu effect of the regional sports industry strong province [58]. It provides the scale, maturity, and financial agglomeration effects [59], possibly due to urbanization and larger space development. Moreover, it could be provoked by the gap between regional urbanization development and the existence of land urbanization, leading to inefficient sports industry economic effects.

Despite the valuable findings, further investigations should consider the efficiency of governance and extend the number of countries for analysis. In addition, innovations boost the development of digital technologies, which could affect the population’s well-being and health.

## Figures and Tables

**Table 1:** Descriptive statistics.

Variable	Mean	Standard Deviation	Minimum	Maximum
Total output value of the sports industry (RMB 100 million yuan)	1270.12	1296.55	15.89	5403
aging of the population (%)	16.42	3.81	9.22	28.1
R & D expenditure (RMB 100 million Yuan)	535.3	551.5	18.6	2499.9
industrial structure	92.1	4.3	84	99.7
Human capital (human beings)	2829.93	763.41	1690	5429
urbanization (%)	62.70	12.5	40.14	89.3
Opening degree (US $10 billion)	194.41	249.68	1.78	1084.46
Transportation Infrastructure (km^2^)	3848.64	1840.07	465	7941

**Table 2:** The findings of the VIF test.

	Ts	Age	Rd	Is	Hc	Urb	Open	Tra
VIF	5.45	2.68	3.77	3.05	1.61	1.78	3.27	4.12
1/VIF	0.183	0.374	0.265	0.328	0.621	0.563	0.305	0.243

**Table 3:** Impact of population aging on the total output value of the sports industry through the technological innovation intermediary path.

Variables	The Control Variables Are Not Considered			Consider Control Variables		
	Sports Industry Development (1)	Technical Innovation (2)	Total Output Value of the Sports Industry (3)	Total Output Value of the Sports Industry (4)	Technical Innovation (5)	Sports Industry Development (6)
Technical innovation			0.016 ***			0.087 ***
Aging of population	0.132 ***	2.356 *	0.094 ***	0.195 ***	3.355 ***	0.098 ***
Industrial structure				0.015 **	0.063 **	0.082 *
Human capital				0.067 *	0.163 *	0.074 *
urbanization				0.042	−0.812	0.048
Open to the outside world				0.028 ***	0.021***	0.011 *
Transportation infrastructure				0.035 ***	0.044 ***	0.037 ***
Constant term	4.318 ***	14.69 *	4.08 ***	0.359 **	−9.358 *	0.432 *
Individual fixed	control	control	control	control	control	control
Time fixed	control	control	control	control	control	control
Sargan test	0.325	0.346	0.250	0.320	0.323	0.335
AR(1)	0.002	0.001	0.000	0.000	0.000	0.000
*p*-value	0.002	0.001	0.000	0.000	0.000	0.000
AR(2)	0.237	0.138	0.145	0.159	0.239	0.215
*p*-value	0.014	0.013	0.032	0.006	0.002	0.023
Adj-R^2^	0.531	0.826	0.543	0.683	0.804	0.702
*p*-value	0.000	0.004	0.000	0.000	0.000	0.000

Note: ***, **, and * are significant at the 1%, 5%, and 10% levels, respectively.

**Table 4:** Results of the robustness test of the intermediary effect of the impact of population aging on the development of the sports industry.

	The Control Variables Are Not Considered		
Variables	Sports Industry Development (7)	Technical Innovation (8)	Sports Industry Development (9)
Technical innovation			0.075 ***
Aging of population	0.165 ***	0.047 **	0.029 ***
Constant term	2.789 ***	6.511 ***	342.0 ***
Individual fixed	control	control	control
Time fixed	control	control	control
Adj-R^2^	0.519	0.663	0.798
*p*-value	0.007	0.02	0.000

Note: *** and **, are significant at the 1%, and 5% levels, respectively.

## Data Availability

Not applicable.
